# Exploring Eating Behaviors and Body Image Perception in Tunisian Women

**DOI:** 10.1192/j.eurpsy.2026.11369

**Published:** 2026-07-31

**Authors:** K. Mahfoudh, A. Hakiri, A. Jaoua, G. Amri, R. Ghachem

**Affiliations:** Department B, Razi Hospital, Manouba, Tunisia

## Abstract

**Introduction:**

Eating habits and body image are two interrelated dimensions with major implications for women’s health. Irregular eating patterns, frequent snacking, or restrictive dieting may contribute to weight fluctuations, while body image concerns are strongly influenced by cultural and social ideals. In Tunisia, young women are increasingly exposed to social media and changing lifestyles, which may foster unhealthy eating behaviors and body dissatisfaction. Despite the growing importance of these issues, few studies have explored them in the Tunisian female context.

**Objectives:**

This study aims to describe the eating habits and body image perception of Tunisian women using validated instruments.

**Methods:**

We conducted a cross-sectional descriptive study from October 1 to November 30, 2024. Participants were Tunisian women aged 18 years and older, recruited online through Facebook, Instagram, and TikTok. Data were collected using a questionnaire in Tunisian dialect, including sociodemographic variables, eating habits (number of meals, snacking, diet history, nutritional follow-up), body mass index (BMI), and psychosocial factors. Body image was assessed with the Arabic validated version of the Body Appreciation Scale-2 (BAS-2).

**Results:**

A total of 365 Tunisian women participated in the study, with a mean age of 33.7 ± 8.9 years (range: 18–66). Most participants (90.0%) had a university-level education.

In terms of lifestyle habits, 36.2% of participants reported practicing regular physical activity. Tobacco use was reported by 14.3% of women, with 46.2% smoking 5–10 cigarettes per day, 30.8% more than 10, and 23.1% fewer than 5. Alcohol consumption concerned 13.4% of participants, mostly occasional (74.0%), while 22.0% reported weekly consumption.

Regarding eating habits, 57.3% reported consuming three main meals per day, 32.9% two meals, 5.2% only one meal, and 4.7% more than three meals. More than half (58.6%) reported snacking between meals. About one-third (35.3%) had consulted a nutritionist for weight management, most often only once (45.9%), while 32.6% were currently following a diet. Among those on a diet, 74.6% reported weight changes, and 68.5% were dieting without professional supervision.

Concerning body image, 65.2% reported a positive or very positive body image, while 11.0% reported a negative perception.

**Conclusions:**

This study provides descriptive insights into the eating habits and body image of Tunisian women, emphasizing the need for culturally sensitive interventions that promote healthy eating and positive body perception.

**Disclosure of Interest:**

None Declared

